# Complete blood count-derived myeloid-lymphoid inflammatory phenotype in rheumatoid arthritis complicated by acute myocardial infarction: an age- and sex-balanced retrospective study

**DOI:** 10.3389/fmed.2026.1905681

**Published:** 2026-09-03

**Authors:** Jing Zhang, Ji Li, Yuwei Wang, Lina Zhang, Di Jin, Sheng-Guang Li, Yuhua Yan

**Affiliations:** 1 Department of Rheumatology and Immunology, Peking University International Hospital, Beijing, China; 2 Department of Emergency Internal Medicine, Weifang People’s Hospital, Weifang, Shandong, China; 3 Department of Rheumatology, Weifang People’s Hospital, Weifang, Shandong, China

**Keywords:** acute myocardial infarction, cardiovascular comorbidity, complete blood count, inflammatory phenotype, myeloid-lymphoid phenotype, neutrophil-to-lymphocyte ratio, rheumatoid arthritis, systemic immune-inflammation index

## Abstract

**Background:**

Rheumatoid arthritis (RA) is associated with excess cardiovascular morbidity, including acute myocardial infarction (AMI). Routine complete blood count (CBC)-derived inflammatory indices are clinically accessible, but their value for characterizing cardiovascular comorbidity in RA remains incompletely defined. We examined whether CBC-derived markers form a coordinated myeloid-lymphoid inflammatory phenotype associated with AMI in patients with RA.

**Methods:**

We retrospectively analyzed 110 healthy controls, 246 consecutive patients with RA without AMI, and 90 consecutive patients with RA and AMI treated at Weifang People's Hospital between January 2020 and December 2025. The primary phenotyping analysis used an age- and sex-balanced sample of 57 participants per group. CBC-derived indices were log2-transformed and standardized. Principal component analysis (PCA), permutational multivariate analysis of variance (PERMANOVA), ordinal logistic regression, RA-only multivariable logistic regression, bootstrap elastic-net stability analysis, and correlation-network mapping characterized CBC-derived inflammatory patterns.

**Results:**

In the full cohort, RA-AMI participants were older and less often female than RA-noAMI and healthy controls. Within RA, AMI was accompanied by longer disease duration, greater glucocorticoid exposure, higher CRP/ESR/DAS28-CRP, higher triglycerides, lower HDL-C, and more frequent cardiometabolic risk. In the matched sample, CBC-derived inflammatory indices increased stepwise from healthy controls to RA without AMI and RA with AMI. The first CBC phenotype principal component (PC1) explained 61.1% of multivariable variance and showed positive loadings for AISI, SIRI, SII, NLR, MLR, neutrophils, monocytes, and platelets, with an opposite loading for lymphocytes. Group-level multivariable separation was confirmed by PERMANOVA (R2 = 0.20, pseudo-F = 20.85, *P* = 0.0002). Each 1-SD increase in PC1 was associated with higher clinical tier (OR 3.64, 95% CI 2.51–5.28). Within RA, PC1 remained associated with AMI status after adjustment for age, sex, glucocorticoid dose, CRP, HDL-C, LDL-C, and cardiometabolic risk (adjusted OR 1.99, 95% CI 1.32–3.02). Bootstrap elastic-net analysis most consistently retained NLR and neutrophil burden.

**Conclusion:**

Routine CBC-derived indices characterize a coordinated myeloid-lymphoid inflammatory phenotype associated with concurrent AMI in RA. These findings support CBC-based inflammatory phenotyping as a low-cost, hypothesis-generating research approach, while direct mechanistic, longitudinal, and prospective validation remains necessary.

## Introduction

Rheumatoid arthritis (RA) is a chronic immune-mediated rheumatic disease defined by persistent synovial inflammation and systemic manifestations (1, 2). Acute myocardial infarction (AMI), defined by contemporary clinical criteria integrating myocardial injury and evidence of ischemia, represents one of the most clinically important cardiovascular events relevant to RA care (3). Patients with RA have an increased risk of incident cardiovascular events and cardiovascular mortality, and international recommendations emphasize systematic cardiovascular risk management in inflammatory joint disease (4–6).

The excess cardiovascular burden in RA is not explained by traditional risk factors alone. Higher RA disease activity and inflammatory burden have been linked to cardiovascular events and myocardial infarction (7, 8). In parallel, lipid-inflammatory interactions, glucocorticoid exposure, and treatment-related pathways may modify cardiovascular risk in RA (9–11). More broadly, atherosclerosis and post-infarction injury are increasingly understood as inflammatory processes, as supported by mechanistic vascular biology and clinical trials of anti-inflammatory therapy in atherosclerotic disease and after myocardial infarction (12–14).

Routine complete blood count (CBC)-derived markers provide a pragmatic way to summarize systemic inflammation. Neutrophil-to-lymphocyte ratio (NLR) and related indices have shown diagnostic or prognostic associations in acute coronary syndromes and AMI, while systemic composite indices such as the systemic immune-inflammation index (SII), systemic inflammation response index (SIRI), and aggregate index of systemic inflammation (AISI) have been increasingly applied across inflammatory and cardiovascular contexts (15–22). In RA, NLR, platelet-to-lymphocyte ratio (PLR), and related composite markers have been associated with disease activity, diagnostic discrimination, subclinical atherosclerosis, and cardiovascular mortality (23–26). RA-specific cardiovascular prediction work further underscores the need to integrate inflammatory and traditional risk domains rather than treating RA as a purely cardiometabolic condition (27).

Despite this background, the CBC phenotype of RA complicated by AMI remains insufficiently characterized. Most RA studies evaluate inflammatory ratios in relation to disease activity or long-term outcomes, whereas AMI studies often focus on post-event prognosis in cardiac cohorts. The present study was therefore designed as a clinical-translational rheumatology phenotyping analysis. We asked whether routinely available CBC variables and derived indices form a coordinated myeloid-lymphoid inflammatory phenotype across healthy controls, RA without AMI, and RA with AMI. We intentionally interpret these data as hypothesis-generating clinical phenotyping rather than direct proof of cellular or molecular mechanisms.

## Methods

### Study design and source populations

This retrospective observational study included all eligible patients with RA evaluated at Weifang People's Hospital between January 2020 and December 2025. Patients with RA, with or without AMI, were consecutively identified from the institutional electronic medical records according to the study definitions; no random sampling was performed. Healthy controls were selected from individuals undergoing routine health examinations at the same hospital during the same period. Consecutive inclusion was used to preserve the temporal continuity of the clinical cohort and to reduce selection arising from discretionary case sampling. RA was classified according to the 2010 ACR/EULAR classification criteria (2). AMI status was identified from clinical records according to contemporary diagnostic practice consistent with the universal definition of myocardial infarction (3).

For participants with RA complicated by AMI, the CBC measurements analyzed were obtained during the AMI-related hospitalization as part of routine clinical evaluation. Because the study was retrospective, the exact interval from symptom onset to blood collection, whether blood was obtained before or after coronary angiography or percutaneous coronary intervention, and the uniformity of sampling conditions could not be consistently reconstructed.

The full cohort included 110 healthy controls, 246 patients with RA without AMI, and 90 patients with RA and AMI. Because the healthy-control source file contained CBC variables and demographics but not the full rheumatology or lipid panel, variables such as BMI, disease duration, RF/anti-CCP status, glucocorticoid exposure, ESR, CRP, DAS28-CRP, and serum lipids were available only within the RA cohort. We retained this structure transparently and did not impute variables absent by design in healthy controls.

### Clinical variables and CBC-derived indices

Baseline variables included age, sex, hemoglobin, white blood cell count, RA duration, body mass index, RF and/or anti-CCP positivity, glucocorticoid use, glucocorticoid dose, ESR, CRP, DAS28-CRP, total cholesterol, triglycerides, HDL-C, LDL-C, and a summary cardiometabolic risk indicator. Current glucocorticoid use was treated as a binary exposure, whereas glucocorticoid dose was analyzed continuously as prednisone-equivalent mg/day.

CBC-derived indices were calculated from routine blood counts as follows: NLR = neutrophils/lymphocytes; PLR = platelets/lymphocytes; MLR = monocytes/lymphocytes; SII = platelets  ×  neutrophils/lymphocytes; SIRI = neutrophils  ×  monocytes/lymphocytes; and AISI = neutrophils  ×  monocytes  ×  platelets/lymphocytes. To reduce right-skew and to support multiplicative interpretation, indices used in modeling were log2-transformed. A composite CBC inflammatory signature was defined as the mean of standardized log2-transformed NLR, MLR, SII, SIRI, and AISI. A myeloid-lymphoid signature was defined from standardized log2-transformed NLR, MLR, and SIRI.

For multivariable CBC phenotyping, raw neutrophil, monocyte, lymphocyte, and platelet counts were also log2-transformed and standardized. This allowed cell counts and derived ratios to enter the same analytic space without dominance by measurement scale. Hemoglobin was retained for baseline description but was not included in the main phenotype feature set because the target of the analysis was leukocyte-platelet inflammatory patterning rather than anemia biology.

### Baseline description and matching strategy

Baseline characteristics were summarized in the full cohort before any matching. Approximately symmetric variables are reported as mean ± standard deviation, whereas skewed variables are reported as median [interquartile range]. Group comparisons for variables available in all three cohorts used one-way ANOVA or Kruskal–Wallis tests as appropriate; categorical variables used chi-square tests. For variables unavailable in healthy controls, *P* values compare RA without AMI with RA with AMI only.

The primary phenotyping analysis used an age- and sex-balanced matched sample with 57 participants in each tier. RA-AMI participants served as anchors, and one healthy control plus one RA-noAMI participant of the same sex and nearest available age were selected without replacement. Only complete three-group triplets were retained, yielding 57 participants per tier. Covariate balance for age and sex was assessed using standardized mean differences (SMDs) before and after matching; an absolute SMD <0.10 was considered indicative of negligible imbalance (28). Pairwise balance diagnostics are presented in Supplementary Table S1. The overall reporting approach followed STROBE principles for observational studies (29).

### CBC phenotype analyses

Principal component analysis (PCA) was applied to standardized log2-transformed NLR, MLR, SII, SIRI, AISI, neutrophils, monocytes, lymphocytes, and platelets in the matched sample. The first principal component (PC1) was interpreted as the dominant CBC-derived myeloid-lymphoid phenotype axis when its loading pattern was biologically coherent and when it showed an ordered relation with clinical tier. The first three components were retained for visualization, but PC1 was the main summar*y* axis because it captured the largest coordinated variance pattern.

To test whether the multivariable CBC phenotype differed across healthy controls, RA without AMI, and RA with AMI, we used PERMANOVA with 4,999 label permutations. We summarized PC1 by group, assessed monotonic trend using the Jonckheere–Terpstra test, and fitted ordinal logistic models with clinical tier ordered as healthy control < RA without AMI < RA with AMI. Each marker was standardized to 1-SD units so that odds ratios could be compared across indices.

Within the RA cohort, multivariable logistic regression modeled AMI status after adjustment for age, sex, glucocorticoid dose, log2(CRP + 1), HDL-C, LDL-C, and cardiometabolic risk. These covariates were selected because they represent clinically plausible confounders linking inflammatory state, treatment exposure, lipid context, and cardiovascular phenotype in RA.

### Feature-stability and network analyses

Because CBC-derived indices are strongly collinear, bootstrap elastic-net logistic regression was used as a stability analysis rather than as a final prediction model. The immune-inflammatory feature set included log2-transformed neutrophils, lymphocytes, monocytes, platelets, NLR, SIRI, the composite CBC signature, and the myeloid-lymphoid signature, while age, sex, glucocorticoid dose, CRP, HDL-C, LDL-C, and cardiometabolic risk entered as adjustment variables. Across 400 bootstrap resamples, we recorded how often each feature retained a non-zero coefficient after penalized adjustment. Selection frequency was interpreted as a stability signal under collinearity, not as proof of unique causal importance.

A correlation-effect network was constructed in the matched sample to visualize the relationship between the main CBC variables and composite indices. Edges represent matched-sample Spearman correlations with |rho| >= 0.55. Node labels show standardized mean differences (Cohen's d based on the pooled standard deviation) for log2-transformed features comparing RA with AMI against RA without AMI in the matched sample. These standardized effects complement the 1-SD-scaled odds ratios used in the regression analyses. The network is descriptive and is not a causal graph.

All continuous features used in PCA, ordinal models, and penalized regression were standardized within the relevant analytic dataset. Missing data were handled by complete-case analysis within each model; no imputation was applied. Analyses were implemented in Python using scipy, scikit-learn, and statsmodels, with two-sided tests throughout.

## Results

### Baseline characteristics in the full cohort

In the full cohort, healthy controls were 63.1 ± 6.5 years old on average, RA-noAMI participants were 61.1 ± 11.0, and RA-AMI participants were 69.7 ± 10.1. The RA-AMI group was also less frequently female (65/90, 72.2%) than healthy controls (96/110, 87.3%) or RA-noAMI participants (224/246, 91.1%) (Table 1). Hemoglobin was lower and white blood cell count was higher across the clinical spectrum. These baseline differences show why crude three-group comparisons alone would be insufficient for interpreting CBC-derived patterns.

**Table 1:** Baseline characteristics of the full cohort.

Characteristic	HC (*n* = 110)	RA-noAMI (*n* = 246)	RA-AMI (*n* = 90)	*P* value
Age, years	63.1 ± 6.5	61.1 ± 11.0	69.7 ± 10.1	<0.001
Female, *n* (%)	96 (87.3%)	224 (91.1%)	65 (72.2%)	<0.001
Hemoglobin, g/L	142.0 [133.2, 151.0]	127.5 [119.0, 138.0]	118.5 [104.0, 131.8]	<0.001
White blood cell count, ×10^9/L	5.7 [4.8, 6.8]	5.9 [4.7, 7.4]	7.7 [6.0, 9.6]	<0.001
RF and/or anti-CCP positive, *n* (%)	—	188 (76.4%)	80 (88.9%)	0.018
Current glucocorticoid use, *n* (%)	—	91 (37.0%)	47 (52.2%)	0.017
BMI, kg/m^2^	—	23.9 ± 3.5	23.1 ± 4.5	0.095
RA duration, months	—	36.0 [12.0, 60.0]	120.0 [84.0, 240.0]	<0.001
Glucocorticoid dose, mg/day (prednisone-eq)	—	0.0 [0.0, 5.0]	5.0 [0.0, 23.8]	<0.001
CRP, mg/L	—	4.5 [1.9, 13.4]	13.3 [5.0, 43.2]	<0.001
ESR, mm/h	—	21.0 [12.0, 42.2]	36.0 [23.0, 83.2]	<0.001
DAS28-CRP	—	3.8 ± 1.2	4.7 ± 1.8	<0.001
Total cholesterol, mmol/L	—	4.9 ± 1.1	4.4 ± 1.4	0.004
Triglycerides, mmol/L	—	1.1 [0.8, 1.5]	1.3 [1.0, 1.8]	0.013
HDL-C, mmol/L	—	1.6 ± 0.4	1.1 ± 0.3	<0.001
LDL-C, mmol/L	—	3.0 ± 0.9	2.8 ± 1.2	0.287
Any cardiometabolic risk factor, *n* (%)	—	28 (11.4%)	62 (68.9%)	<0.001

Values are mean ± SD or median [IQR]. “—” indicates not available in the healthy-control source dataset. For RA-only variables, *P* values compare RA-noAMI vs. RA-AMI.

Within the RA cohort, AMI was accompanied by a more inflammatory and comorbidity-enriched phenotype. RA-AMI participants had longer disease duration, greater glucocorticoid exposure, higher CRP, ESR, and DAS28-CRP, higher triglycerides, lower HDL-C, and substantially more frequent cardiometabolic risk factors than RA-noAMI participants (Table 1). These findings are consistent with clinical overlap between RA inflammatory burden, treatment exposure, lipid context, and cardiovascular comorbidity.

### Stepwise CBC-derived inflammatory phenotype in the matched sample

**Figure 1:**
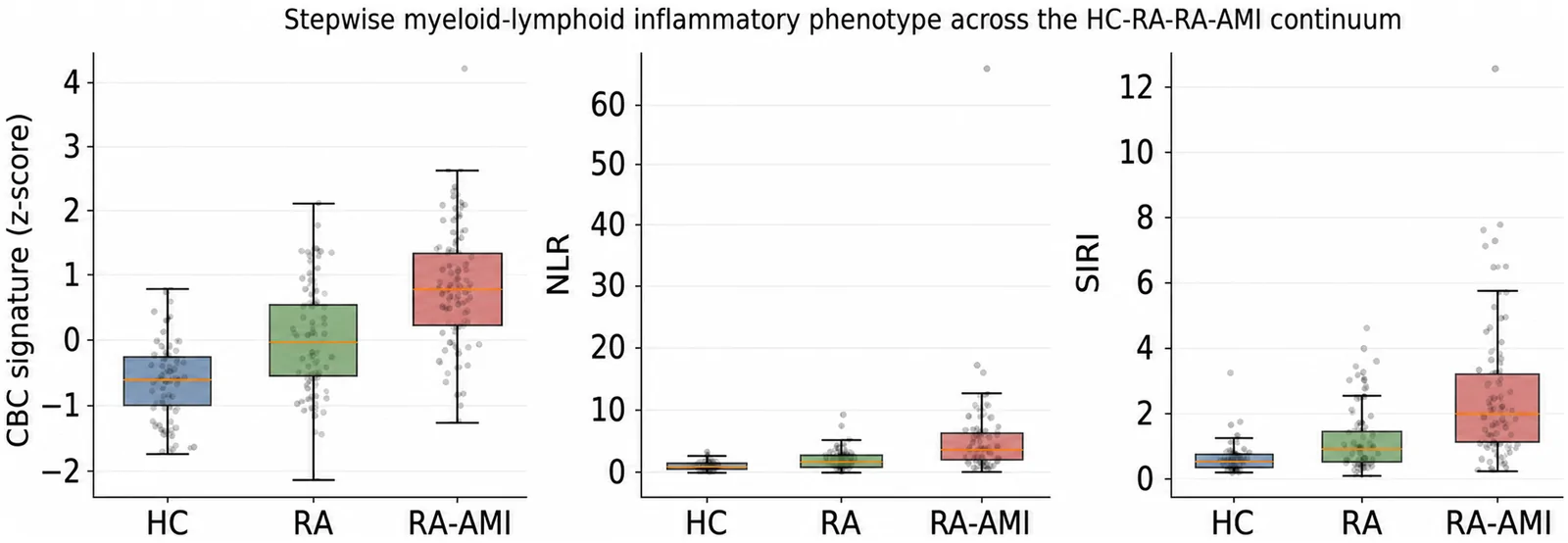
Stepwise CBC-derived inflammatory phenotype across healthy controls, RA without AMI, and RA with AMI. Boxplots show the composite CBC signature, NLR, and SIRI in the age- and sex-balanced matched sample.

The primary matched analysis included 57 participants in each tier. Sex distribution was identical across groups by design, and all pairwise absolute SMDs for age were below 0.10 after matching, compared with substantially larger imbalances in the full cohort (Supplementary Table S1). Representative CBC-derived indices showed a stepwise increase from healthy controls to RA without AMI and RA with AMI (Figure 1). This pattern was observed after age and sex balancing, indicating that the inflammatory gradient was not solely explained by the major demographic imbalance present in the full cohort.

**Figure 2:**
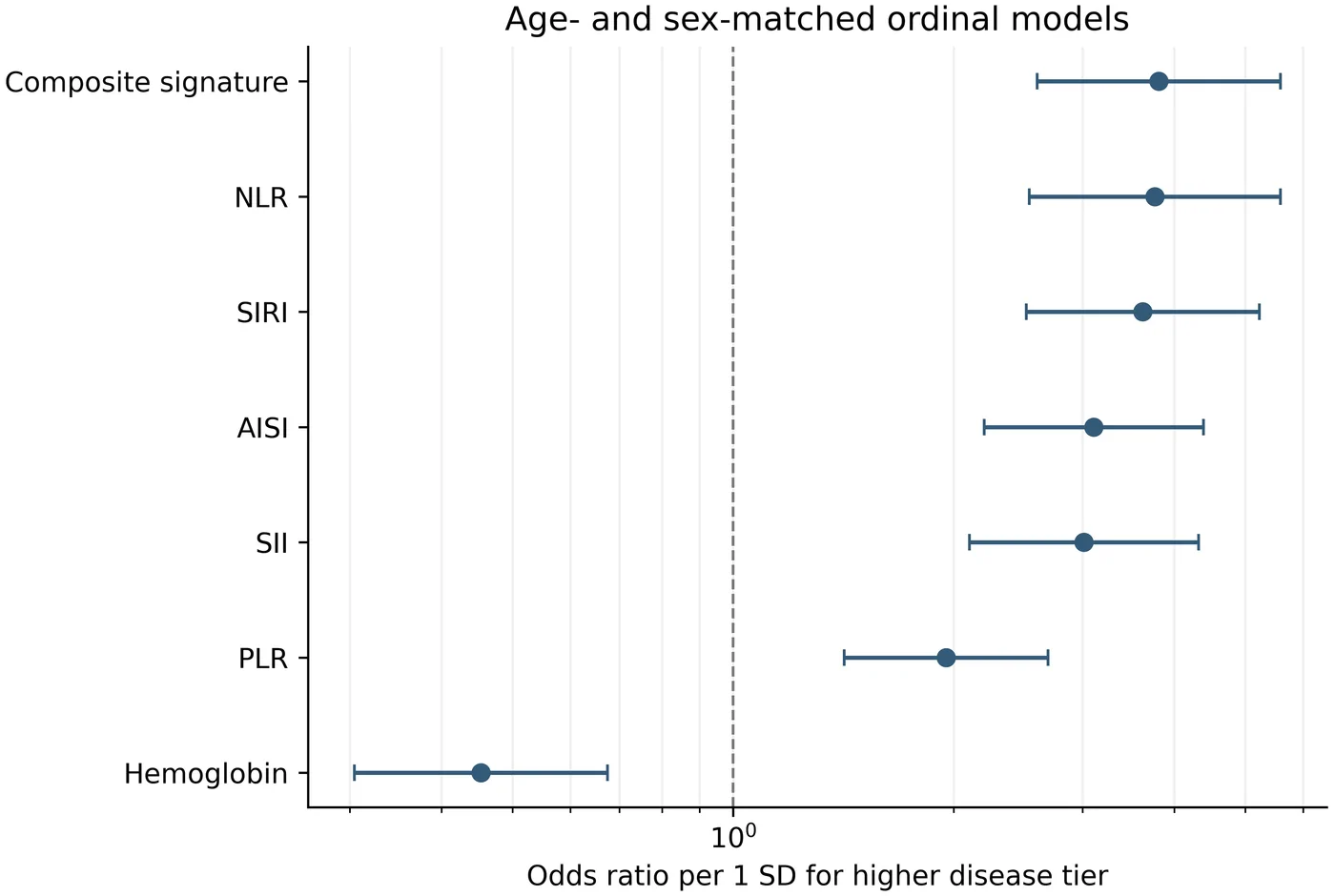
Age- and sex-matched ordinal logistic regression models. Odds ratios are per 1-SD increase and estimate the odds of membership in a higher clinical tier.

Matched ordinal logistic models confirmed strong associations between standardized CBC-derived markers and higher clinical tier (Table 3**;** Figure 2). The composite CBC signature, NLR, SIRI, AISI, and SII all showed large effect estimates per 1-SD increase, whereas PLR showed a more moderate but still statistically significant association. These conventional analyses provide the empirical basis for treating the CBC data as a coordinated inflammatory phenotype rather than as isolated ratios.

### PCA identifies a dominant myeloid-lymphoid CBC phenotype axis

PCA of standardized log2-transformed CBC variables and derived indices identified a dominant first component explaining 61.1% of multivariable variance. PC1 showed positive loadings for AISI, SIRI, SII, NLR, MLR, neutrophils, monocytes, and platelets, and an opposite loading for lymphocytes (Table 2). This loading pattern supports interpretation of PC1 as a myeloid-rich, lymphoid-low CBC inflammatory phenotype axis.

**Table 2:** PCA loadings for the CBC-derived phenotype in the matched sample.

Feature	PC1 loading	PC2 loading
NLR	0.388	−0.251
MLR	0.345	−0.214
SII	0.394	−0.049
SIRI	0.416	0.021
AISI	0.417	0.153
NEU	0.340	0.278
MONO	0.219	0.427
LYMPH	−0.204	0.653
PLT	0.152	0.423

Positive PC1 values represent higher myeloid-composite inflammatory loading and lower relative lymphoid contribution.

**Table 3:** Matched ordinal models for higher clinical tier.

Marker	OR per 1-SD for higher tier	P
NLR	3.76 (2.54–5.59)	<0.001
PLR	1.95 (1.42–2.69)	<0.001
SII	3.01 (2.10–4.32)	<0.001
SIRI	3.62 (2.51–5.23)	<0.001
AISI	3.11 (2.20–4.39)	<0.001
CBC signature	3.81 (2.60–5.59)	<0.001

Ordinal logistic regression used the age- and sex-balanced matched sample and ordered the clinical tier as healthy control < RA without AMI < RA with AMI. Odds ratios are per 1-SD increase.

Median PC1 increased from −1.50 in healthy controls to −0.08 in RA without AMI and 1.89 in RA with AMI, with a significant ordered trend (Jonckheere–Terpstra *P* < 0.001). In the PCA plane, group centroids moved progressively along the same direction (Figure 4). PERMANOVA confirmed group-level multivariable separation (R2 = 0.20, pseudo-F = 20.85, *P* = 0.0002). Each 1-SD increase in PC1 was associated with higher clinical tier (OR 3.64, 95% CI 2.51–5.28, *P* < 0.001).

### RA-only adjusted models and feature stability

**Figure 3:**
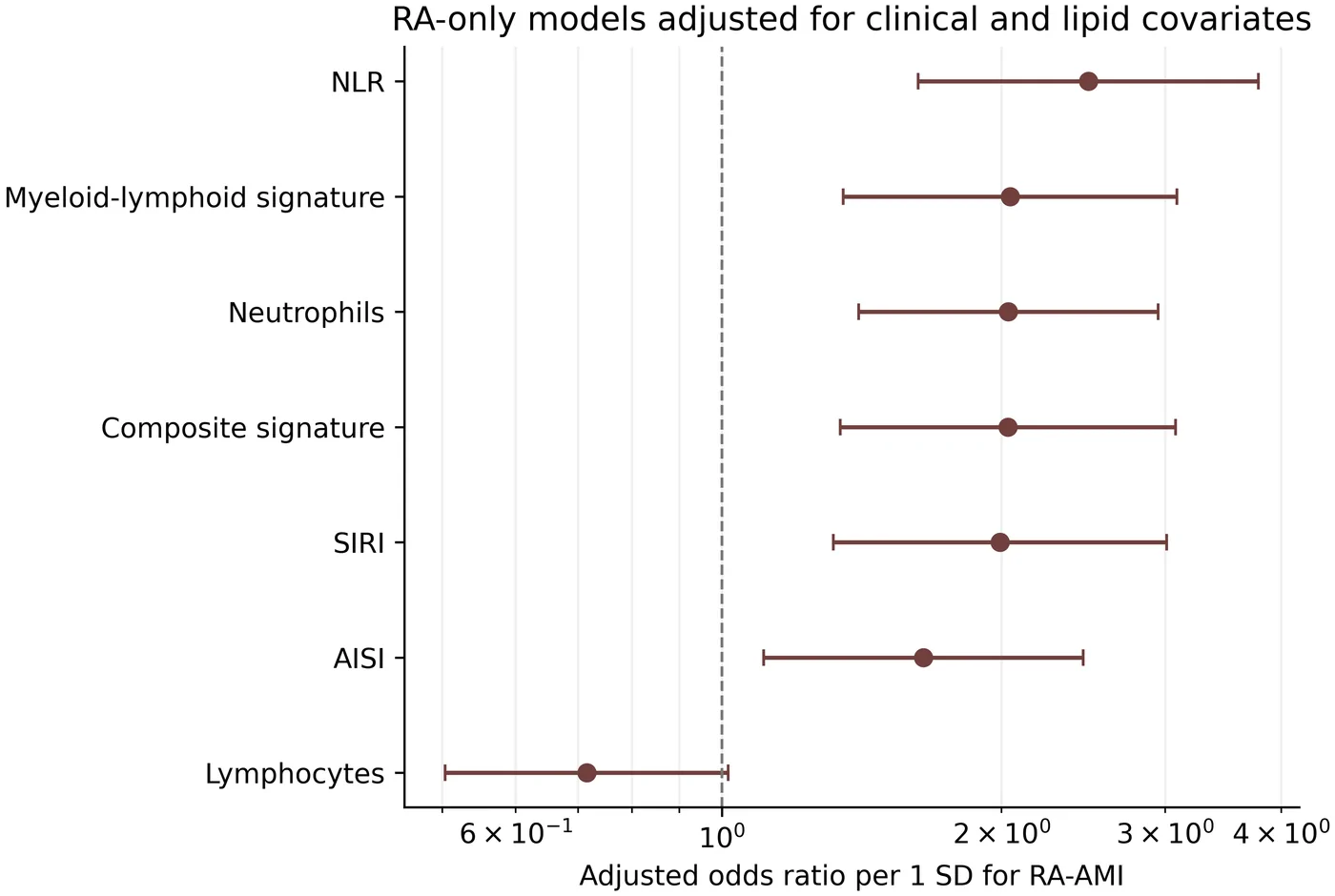
RA-only adjusted logistic regression models for AMI status. Each marker was entered separately and adjusted for age, sex, glucocorticoid dose, log2(CRP + 1), HDL-C, LDL-C, and cardiometabolic risk factors.

Within RA, CBC phenotype PC1 remained associated with AMI status after adjustment for age, sex, glucocorticoid dose, log2(CRP + 1), HDL-C, LDL-C, and cardiometabolic risk (adjusted OR 1.99, 95% CI 1.32–3.02, *P* = 0.001). RA-only adjusted models of individual markers similarly showed that neutrophil-rich and composite myeloid-lymphoid indices were associated with AMI status (Figure 3).

**Figure 4:**
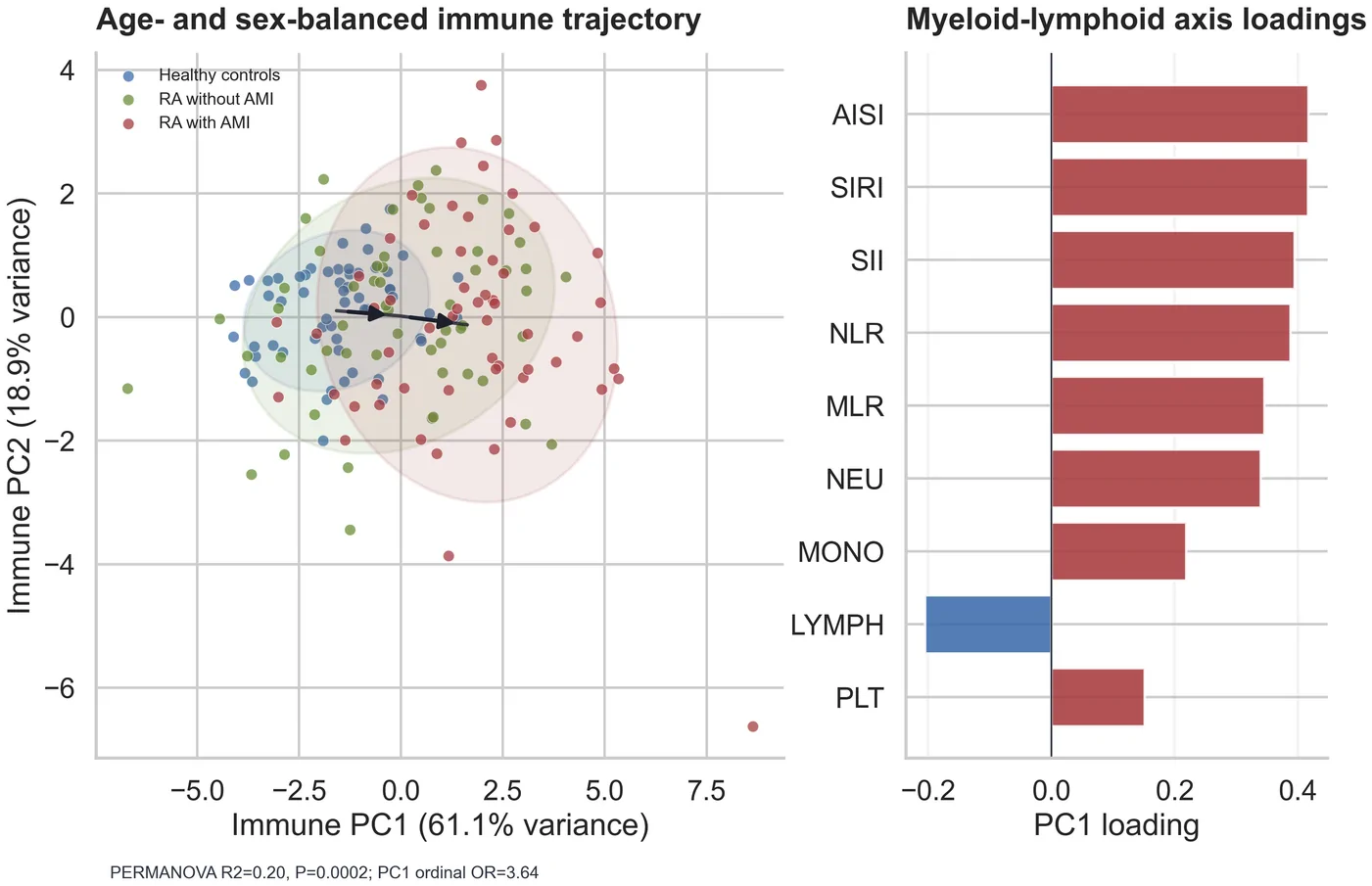
Age- and sex-balanced CBC-derived inflammatory phenotype derived from PCA of log2-transformed CBC variables and indices. PC1 captures the dominant myeloid-lymphoid phenotype axis.

**Figure 5:**
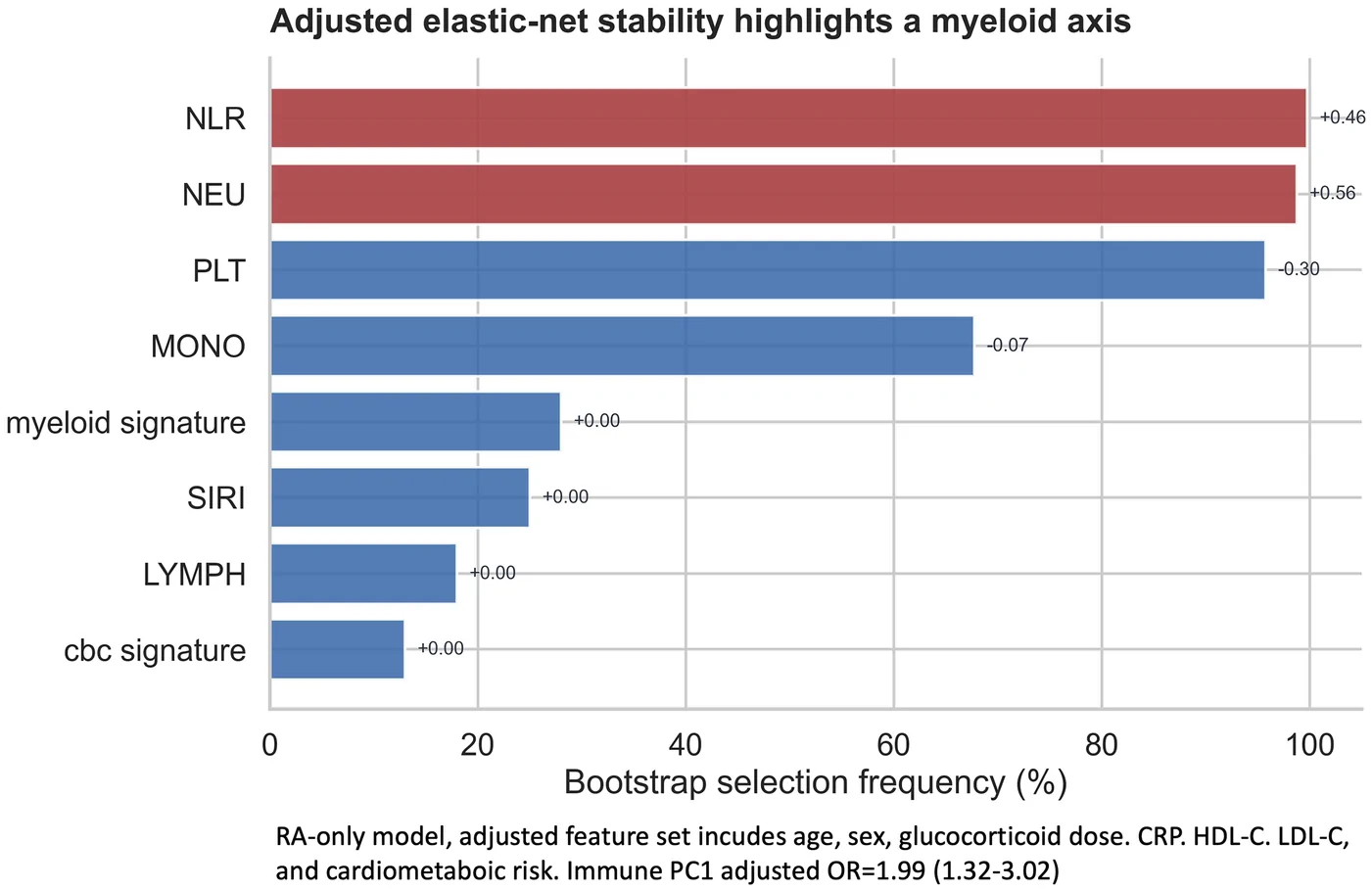
Bootstrap elastic-net stability analysis in the RA cohort after inclusion of clinical covariates. Selection frequencies are exploratory stability signals and should be interpreted in the context of collinearity.

Bootstrap elastic-net stability analysis then evaluated which variables survived repeated penalized adjustment in a collinear feature space. NLR and neutrophil burden were retained in 99.8% and 98.8% of bootstrap resamples, respectively (Table 4; Figure 5). Platelet count was also frequently retained, but with a conditional negative median coefficient after simultaneous adjustment, suggesting that platelet information in this dataset may be context-dependent and should not be interpreted as a simple standalone pro-AMI marker. Higher-order summary signatures were selected less consistently after their shared variance was decomposed into constituent cell-count and ratio features.

**Table 4:** Bootstrap elastic-net feature stability in RA.

Feature	Bootstrap selection frequency	Median standardized coefficient
log2_NLR	99.8%	+0.458
log2_NEU	98.8%	+0.556
log2_PLT	95.8%	−0.297
log2_MONO	67.8%	−0.071
myeloid-lymphoid signature	28.0%	+0.000
log2_SIRI	25.0%	+0.000
log2_LYMPH	18.0%	+0.000
CBC signature	13.0%	+0.000

Bootstrap elastic-net logistic regression was performed in the RA cohort with clinical covariate adjustment. Selection frequencies are stability signals in a collinear feature space, not evidence of unique causal effects.

**Figure 6:**
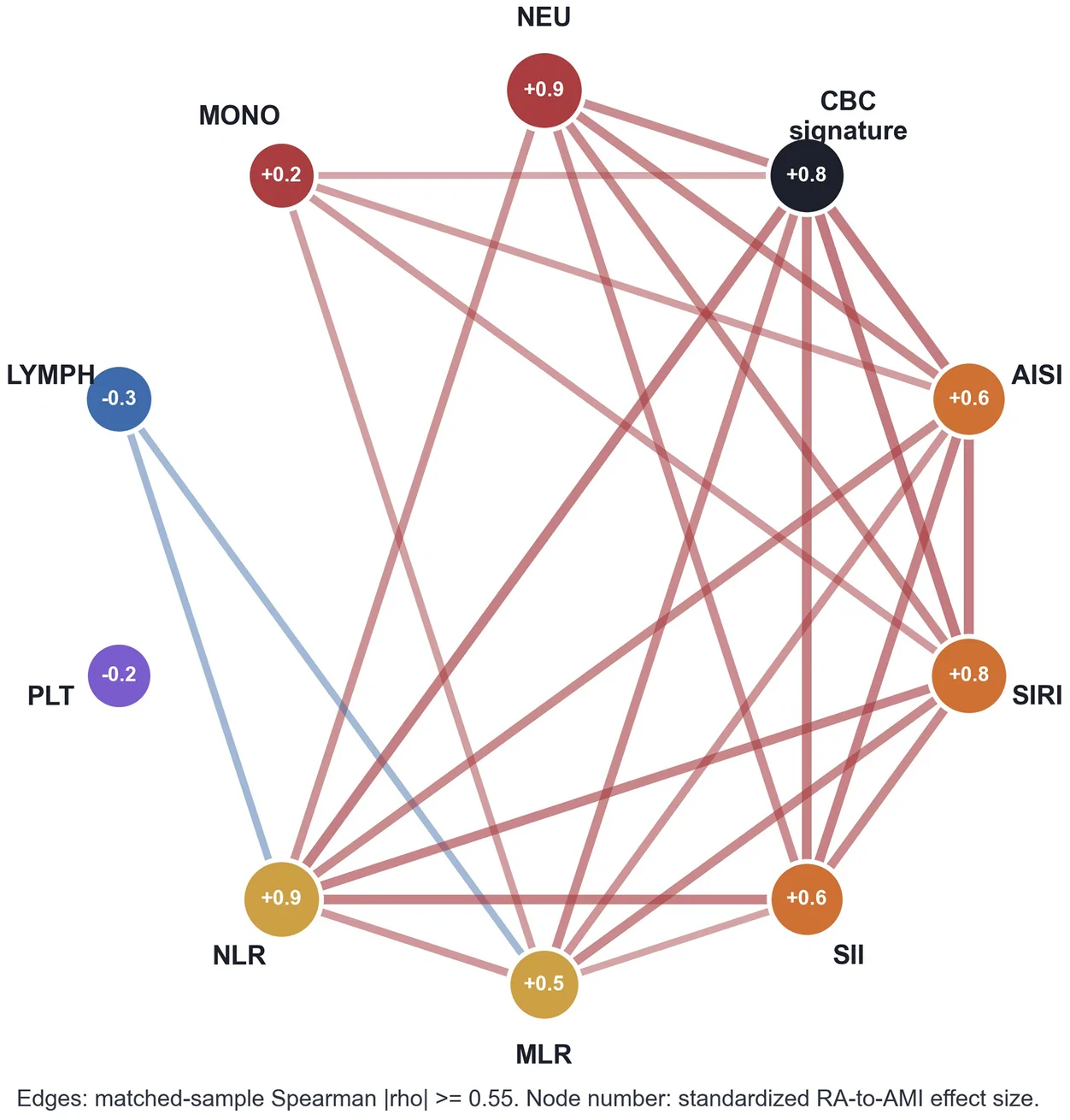
Correlation-effect network of the CBC-derived inflammatory phenotype. Edges indicate matched-sample Spearman correlations with |rho| >= 0.55; node labels show standardized RA-to-AMI effect sizes.

The correlation-effect network showed a dense positive module involving neutrophils, NLR, SIRI, SII, AISI, and the composite CBC signature, whereas lymphocyte measures occupied an opposing direction (Figure 6). In the matched RA comparison, standardized effects were largest for neutrophil count and NLR (approximately 0.9 SD), intermediate for SIRI, SII, AISI, and MLR, and smaller or negative for lymphocyte and platelet counts. This descriptive network reinforces the PCA and elastic-net findings: the observed signal is best understood as a coordinated CBC-derived inflammatory phenotype centered on neutrophil-rich myeloid loading and relative lymphoid attenuation.

## Discussion

### Principal findings

This retrospective clinical-translational rheumatology study shows that routinely available CBC-derived indices form a coordinated myeloid-lymphoid inflammatory phenotype associated with AMI in patients with RA. The main contribution is not direct proof of immunothrombotic mechanisms, but rather the identification of a low-cost inflammatory phenotype that characterizes RA complicated by AMI after age and sex balancing and after RA-only clinical adjustment.

Four findings support this interpretation. First, RA-AMI participants had a clinically more inflammatory and comorbidity-enriched profile in the full cohort. Second, in an age- and sex-balanced matched sample, CBC-derived indices increased stepwise across healthy controls, RA without AMI, and RA with AMI. Third, PCA condensed correlated CBC variables into a dominant myeloid-rich, lymphoid-low phenotype axis that separated the three clinical states. Fourth, bootstrap elastic-net analysis indicated that NLR and neutrophil burden were the most stable features after collinearity and clinical adjustment were considered.

### Relevance to cardiovascular comorbidity in RA

Cardiovascular comorbidity remains a major clinical challenge in RA. Traditional cardiovascular risk management is essential, but RA disease activity, chronic inflammation, lipid-inflammatory interactions, and treatment exposures all influence cardiovascular phenotype. The present findings are consistent with this clinical reality: RA-AMI was not merely a group with higher single inflammatory ratios, but a group in which multiple CBC-derived indices aligned into a broader myeloid-lymphoid pattern.

The practical value of this approach is that CBC data are inexpensive, widely available, and routinely collected in rheumatology practice. A CBC-derived inflammatory phenotype should not replace established cardiovascular risk assessment, lipid management, or disease activity control. Instead, it may provide an inexpensive descriptive layer for characterizing RA patients with prominent systemic inflammatory and cardiovascular comorbidity signals and for prioritizing questions for more detailed clinical or mechanistic evaluation. It should not be used to infer future AMI risk from the present cross-sectional data.

Recent studies have extended routine blood-based composite phenotyping to acute coronary syndrome and acute pericarditis using the pan-immune-inflammation value and the hemoglobin-albumin-lymphocyte-platelet score, and to critical illness using combined hematologic and metabolic indices (30–32). Although these populations and endpoints differ from the present study and do not validate an RA-specific phenotype, they illustrate broader translational interest in extracting clinically interpretable information from routinely collected laboratory variables.

### Biological interpretation and boundaries

The observed phenotype is biologically plausible but must be interpreted cautiously. Neutrophils have recognized roles in rheumatic disease-associated vascular inflammation, while RA-related atherosclerosis involves cytokine signaling, endothelial dysfunction, monocyte-macrophage biology, lipid-inflammatory interactions, and treatment-related influences (33, 34). Neutrophil extracellular traps, platelet-endothelial crosstalk, and thromboinflammatory pathways have also been implicated in cardiovascular disease and AMI (35–37). These pathways provide a rationale for why a neutrophil-rich and lymphoid-low CBC pattern might track cardiovascular complications in RA.

**Figure 7:**
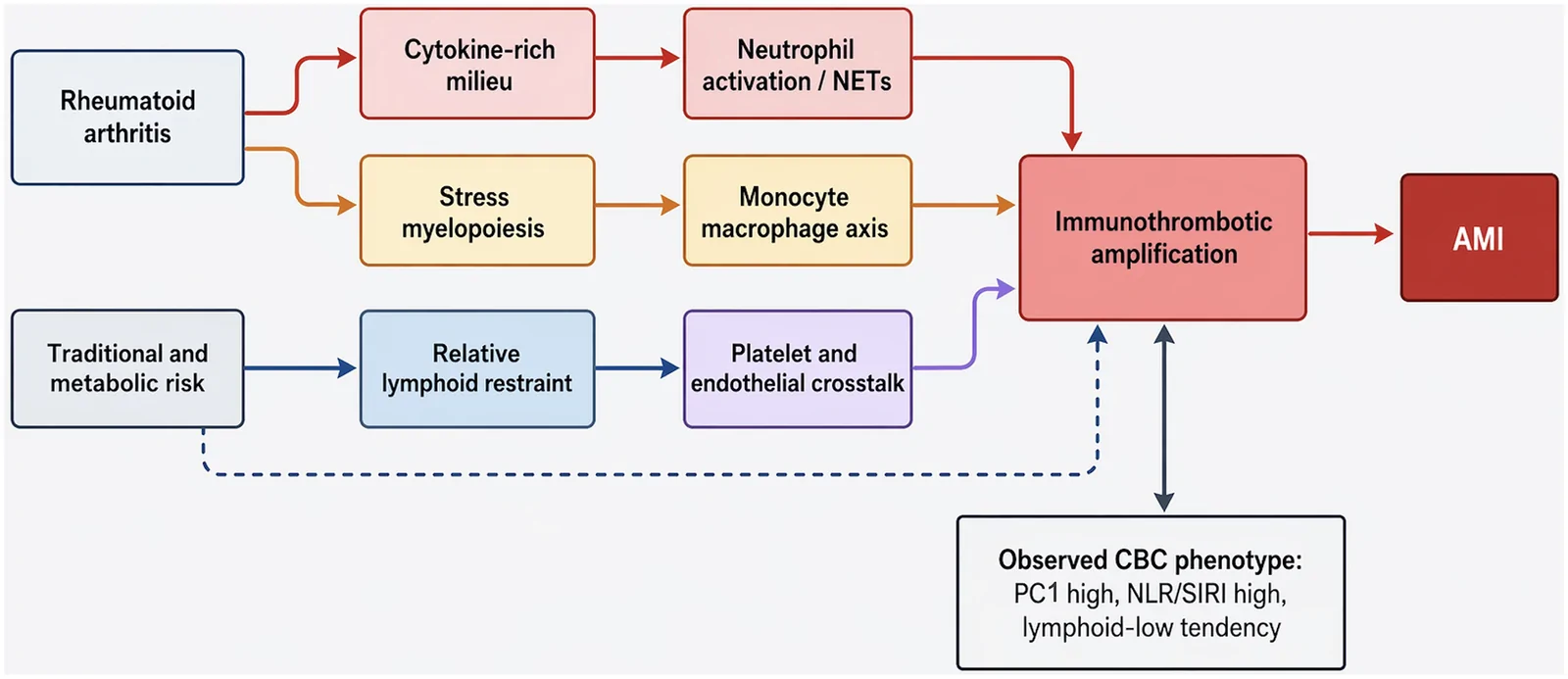
Working hypothesis linking RA inflammatory burden, CBC-derived myeloid-lymphoid phenotype, and AMI. This model is hypothesis-generating and does not demonstrate direct causality, because NET formation, cytokines, endothelial activation, platelet activation, coronary anatomy, and plaque features were not directly measured.

However, the present study did not directly measure NET formation, cytokines, endothelial activation, platelet activation, plaque composition, coronary anatomy, infarct size, or immune-cell subsets. Therefore, the CBC phenotype should be described as compatible with thromboinflammatory biology, not as evidence that these mechanisms were directly demonstrated. Figure 7 presents a working hypothesis for future validation rather than a causal pathway proven by the present data.

### Why multivariable phenotyping was useful

CBC-derived indices are mathematically and biologically collinear. Presenting NLR, SIRI, AISI, SII, neutrophils, monocytes, platelets, and composite scores as independent discoveries would overstate the evidence. The PCA and elastic-net analyses were therefore useful because they made the shared structure explicit. PCA showed that much of the signal was organized around a dominant myeloid-rich, lymphoid-low axis, while elastic-net analysis showed that NLR and neutrophil burden remained most stable when related variables were forced into direct competition.

PC1 should not be interpreted as a clinical prediction score or as superior to NLR. In fact, NLR and neutrophil count were the most stable features in the elastic-net analysis. The role of PC1 in this study was to provide a dimensional summary of correlated CBC information and to demonstrate that the RA-AMI pattern was not restricted to a single ratio. Whether PC1 offers incremental value beyond NLR, SII, and established cardiovascular variables cannot be determined from this cross-sectional dataset. Future studies would require longitudinal outcomes, external validation, and formal assessment of discrimination, calibration, and clinical utility before incorporating such a phenotype into a prediction model.

### Clinical implications and future directions

From a clinical perspective, the current findings support further evaluation of CBC-derived inflammatory phenotyping as a low-cost adjunct to, not a replacement for, guideline-based cardiovascular assessment in RA. At present, the phenotype should be regarded as descriptive and hypothesis-generating. A realistic next step is a prospective study using CBC samples obtained before cardiovascular events to test whether NLR alone, or a parsimonious multivariable phenotype, adds incremental information to established RA and cardiovascular risk factors. Only after external validation could such an approach be considered for flagging patients who may warrant closer cardiovascular review.

### Methodological constraints and interpretation boundaries

Several limitations are inherent to the dataset. First, the analysis is retrospective and cross-sectional with respect to AMI. CBC samples for RA-AMI participants were obtained during the AMI-related hospitalization, and exact symptom-to-sampling intervals and timing relative to coronary intervention were not consistently available. Acute ischemia, stress leukocytosis, hospitalization context, and treatment may therefore contribute substantially to the observed phenotype. The data cannot determine whether this phenotype preceded AMI, and it should be interpreted as AMI-associated rather than predictive of a future event. Second, there was no non-RA AMI comparison group. Consequently, the study cannot distinguish an RA-specific AMI phenotype from the general hematologic inflammatory response accompanying AMI; this is a central limitation of the study. Third, healthy controls lacked the full RA covariate and lipid panel, limiting three-group multivariable adjustment.

Fourth, glucocorticoid exposure may influence neutrophil and lymphocyte counts despite adjustment for dose in RA-only models. Fifth, detailed cardiovascular severity data—including STEMI versus NSTEMI classification, infarct location, peak troponin concentrations, coronary angiographic findings, PCI characteristics, left ventricular ejection fraction, and in-hospital outcomes—were not consistently available and could not be incorporated. Sixth, infection status, medication timing, and other acute-care factors could not be fully resolved. Seventh, CBC-derived indices are highly collinear, so feature-selection findings should be interpreted as stability signals rather than evidence of unique causal effects. Eighth, the matched sample improved age and sex balance but reduced sample size and cannot eliminate residual confounding.

These constraints define the evidentiary level of the study. The findings support a hypothesis-generating CBC-based inflammatory phenotype associated with concurrent AMI in RA; they do not establish causality, prospective prediction, or mechanism. Prospective longitudinal studies with pre-AMI sampling, non-RA AMI comparators, adjudicated cardiovascular events, detailed cardiovascular severity measures, vascular imaging, cytokine panels, NET markers, platelet activation markers, endothelial injury markers, and immune-cell subset profiling are required.

## Conclusion

Routine CBC-derived indices characterize a coordinated myeloid-lymphoid inflammatory phenotype associated with concurrent AMI in RA. In an age- and sex-balanced sample, RA complicated by AMI occupied the high end of a neutrophil-rich, composite-index-high, lymphoid-low CBC phenotype axis. These findings support further study of CBC-based inflammatory phenotyping as a low-cost clinical-translational research approach, but not as a validated predictor. Direct mechanistic and prospective validation is required before causal or clinical decision-making claims can be made.

## Data Availability

The datasets analyzed during the current study are available from the corresponding author on reasonable request, subject to institutional and ethical restrictions.
