# Supplementary material for: Complete blood count-derived myeloid-lymphoid inflammatory phenotype in rheumatoid arthritis complicated by acute myocardial infarction: an age- and sex-balanced retrospective study
Source: Front Med (Lausanne). 2026 Sep 3;13:1905681. doi: 10.3389/fmed.2026.1905681 (PMC13581526; doi:10.3389/fmed.2026.1905681)
Supplement: Supplementary file 1 [file Table1.docx]

**Supplementary Material**

**Complete blood count-derived myeloid-lymphoid inflammatory phenotype in rheumatoid arthritis complicated by acute myocardial infarction: an age- and sex-balanced retrospective study**

**Supplementary Table S1. Standardized mean differences for the matching variables before and after matching**

| **Variable** | **Pairwise comparison** | **Full cohort \|SMD\|** | **Matched sample \|SMD\|** |
| --- | --- | --- | --- |
| Age | HC vs RA-noAMI | 0.219 | 0.041 |
| Age | HC vs RA-AMI | 0.784 | 0.035 |
| Age | RA-noAMI vs RA-AMI | 0.818 | 0.006 |
| Female sex | HC vs RA-noAMI | 0.122 | 0.000 |
| Female sex | HC vs RA-AMI | 0.381 | 0.000 |
| Female sex | RA-noAMI vs RA-AMI | 0.502 | 0.000 |

**Note.** Values are absolute standardized mean differences. An absolute SMD <0.10 was prespecified as indicating negligible imbalance. Matching was performed on age and sex; inflammatory variables were study phenotypes and were therefore not balance targets. HC, healthy controls; RA-noAMI, rheumatoid arthritis without acute myocardial infarction; RA-AMI, rheumatoid arthritis with acute myocardial infarction; SMD, standardized mean difference.
